# Conserved Genome Organization and Core Transcriptome of the *Lactobacillus acidophilus* Complex

**DOI:** 10.3389/fmicb.2018.01834

**Published:** 2018-08-13

**Authors:** Alexandra B. Crawley, Rodolphe Barrangou

**Affiliations:** ^1^Genomic Sciences Program, NC State University, Raleigh, NC, United States; ^2^Department of Food, Bioprocessing and Nutrition Sciences, NC State University, Raleigh, NC, United States

**Keywords:** *Lactobacillus*, core genome, core transcriptome, microbial genetics, chromosome organization

## Abstract

The *Lactobacillus* genus encompasses a genetically and functionally diverse group of species, and contains many strains widely formulated in the human food supply chain as probiotics and starter cultures. Within this genetically expansive group, there are several distinct clades that have high levels of homology, one of which is the *Lactobacillus acidophilus* group. Of the uniting features, small genomes, low GC content, adaptation to dairy environments, and fastidious growth requirements, are some of the most defining characteristics of this group. To better understand what truly links and defines this clade, we sought to characterize the genomic organization and content of the genomes of several members of this group. Through core genome analysis we explored the synteny and intrinsic genetic underpinnings of the *L. acidophilus* clade, and observed key features related to the evolution and adaptation of these organisms. While genetic content is able to provide a large map of the potential of each organism, it does not always reflect their functionality. Through transcriptomic data we inferred the core transcriptome of the *L. acidophilus* complex to better define the true metabolic capabilities that unite this clade. Using this approach we have identified seven small ORFs that are both highly conserved and transcribed in diverse members of this clade and could be potential novel small peptide or untranslated RNA regulators. Overall, our results reveal the core features of the *L. acidophilus* complex and open new avenues for the enhancement and formulation and of next generation probiotics and starter cultures.

## Introduction

With the rise of modern genomics, studies have shed light on the non-random organization of the prokaryotic genome (Rocha, 2008; Touchon and Rocha, 2016). The most striking organizational pattern in the prokaryotic chromosome is the division of the two replichores, or replication arms. Replication in prokaryotes begins at the origin of replication then proceeds toward the terminus, in a bi-directional manner, resulting in two halves of the genome called replichores (Rocha, 2008; Touchon and Rocha, 2016). In bacteria, there is a very heavy strand-bias in density of coding genes between replichores with the majority of the coding sequences arranged on the leading strand where replication occurs in the 5^′^ to 3^′^ direction (Lobry, 1996; Lobry and Lobry, 1999; Frank and Lobry, 2000). Fewer coding sequences are found on the lagging strand where replication occurs 3^′^ to 5^′^ (Lobry, 1996; Lobry and Lobry, 1999). This disparity in coding densities also leads to skews in GC and TA content between the leading and lagging strands which can be used to detect the terminus in prokaryotic genomes (Huynen and Snel, 2000; Rocha, 2008). Importantly, this enables co-directionality of arguably the two most critical biological processes, namely replication and transcription. Other intrinsic genetic factors in prokaryotes, such as codon usage and codon adaptation, are used to determine gene stability and evolutionary divergence of chromosomal regions (Touchon and Rocha, 2016).

Synteny is the conservation of gene order within bacterial chromosomes (Huynen and Snel, 2000; Huynen et al., 2000; Touchon and Rocha, 2016). High levels of synteny are found within bacterial species and highlight patterns of genome stability and conservation in prokaryotes (Rocha, 2008). With the increasing availability of expression data, co-regulation patterns of conserved genes and synteny have been observed in several organisms (Bouyioukos et al., 2016). These patterns have been used to propose models of bacterial evolution, wherein conserved genome layout and gene regulation are inter-dependent and have increased genome stability and conserved 3-dimensional chromosome conformation over time (Huynen and Snel, 2000; Huynen et al., 2000; Bouyioukos et al., 2016).

Lactic acid bacteria (LAB) are a diverse group of Gram-positive, fastidious, non-sporulating *Firmicutes* that produce lactic acid as their major fermentation by-product (Kandler and Weiss, 1986; Axelsson, 2004; Kilian, 2005; Makarova et al., 2006; Sun et al., 2015). The genus *Lactobacillus* is a major constituent of the LAB group which contains the same amount of genetic diversity as many prokaryotic families (Kant et al., 2011; Sun et al., 2015). In order to reduce complexity in lactobacilli, investigators have parsed the genus into phylogenetic clades based on environment and genetic similarity (Thompson and Collins, 1991; Canchaya et al., 2006; Sun et al., 2015). One clade, the *Lactobacillus acidophilus* complex, contains around 17 species, including: *L. acidophilus*, *Lactobacillus amylovorus*, *Lactobacillus helveticus*, *Lactobacillus crispatus*, and *Lactobacillus delbrueckii*. Many of these organisms provide demonstrated benefits to human health as probiotics and are used as industrial dairy starter cultures (Bernardeau et al., 2006; Klaenhammer et al., 2012). The *L. acidophilus* complex has had a tremendous impact on biotechnology, despite the extreme diversity genetically and physiologically (Kullen et al., 2001). Thus, there is a need to better understand the unifying factors of this clade from a genetic and functionality standpoint. Understanding the intrinsic genetic factors that are unique to various members of this clade and common across all organisms can help shed light on best approaches for improvement of strain development as well as means of exploiting these strains for biotechnological purposes. Extending our knowledge of prokaryotic genetics to non-model organisms, like lactobacilli, bolsters our understanding of microbial evolution and conservation, and will provide a molecular basis for their enhancement and the formulation and engineering of next generation probiotics and starter cultures.

With the abundance of genetic data rapidly being generated, there now exists a great opportunity to define intrinsic factors that unite specific prokaryotic clades, like the *L. acidophilus* complex, and determine what unique genomic characteristics set them apart from others. Here, we analyze factors such as GC content, genomic synteny, and gene expression to better understand the *L. acidophilus* clade. While most genetic studies focus solely on gene content, here, we overlay gene presence and absence with expression to better inform how cellular functions may be conserved across this clade. We examine a set of highly conserved and highly expressed genes, called the core transcriptome, in an effort to move beyond cataloging genetic similarities to begin to compare conserved biological functions in the *L. acidophilus* complex.

## Materials and Methods

### Genomics

Six model lactobacilli with fully sequenced, closed genomes derived from reference strains were selected for this study: *Lactobacillus acidophilus* NCFM, *Lactobacillus amylovorus* GRL 112, *Lactobacillus crispatus* ST1, *Lactobacillus delbrueckii subsp. bulgaricus* ATCC 11842, *Lactobacillus gasseri* ATCC33323, and *Lactobacillus helveticus* CRNZ32 (**Table 1**). The terminus and GC/TA skew for each genome was performed using oriloc (Frank and Lobry, 2000), an R (R Core Team, 2017) package. EMBOSS (Rice et al., 2000) was used to calculate the GC content and codon adaptation indices for all coding sequences. The core genome was elucidated as follows: complete genomes were downloaded from NCBI and re-annotated with the PROKKA (Seemann, 2014) prokaryotic genome tool with default parameters to ensure an identical gene detection algorithm was utilized. Roary (Page et al., 2015) was then applied to detect gene presence and absence. To determine a reliable standardized functional gene annotation, the RAST Server (Aziz et al., 2008) was used to functionally annotate the genes.

**Table 1:** Genome summary.

Organism	Total coding genes	Genome size (bp)	%GC	Location of	Genes on right	Genes on right	Genes on left	Genes on left	Genes on forward	Genes on reverse	Reference
				terminus start (bp)	replichore, forward strand	replichore, reverse strand	replichore, reverse strand	replichore, forward strand	strand	strand	
*Lactobacillus acidophilus* NCFM	1834	1993560	34.7	1106894	821 (44.7%)	230 (12.6%)	183 (10.0%)	600 (32.7%)	1421 (77.4%)	413 (22.5%)	Altermann et al., 2005
*Lactobacillus amylovorus* GRL 1112	1696	2067702	38.2	1211255	766 (45.1%)	247 (14.6%)	185 (10.9%)	498 (29.3%)	1264 (74.5%)	432 (25.5%)	Broadbent et al., 2013
*Lactobacillus delbrueckii subsp. bulgaricus* ATCC 11842	1549	1864998	49.7	915000	632 (40.8%)	151 (9.7%)	185 (11.9%)	581 (37.5%)	1173 (78.4%)	346 (21.6%)	Cato et al., 1983; Ojala et al., 2010
*Lactobacillus crispatus* ST1	1939	2043161	36.9	1139949	849 (43.8%)	254 (13.1%)	193 (9.9%)	643 (33.1%)	1492 (77.0%)	447 (23.0%)	Nakamura, 1981; Kant et al., 2011
*Lactobacillus gasseri* ATCC33323	1772	1894360	35.3	1004754	788 (44.4%)	174 (9.8%)	224 (12.6%)	586 (33.1%)	1374 (77.6%)	398 (22.4%)	Johnson et al., 2015
*Lactobacillus helveticus* CNRZ32	1704	2225962	36.9	1329141	766 (44.9%)	234 (13.7%)	136 (8.0%)	568 (33.3%)	1334 (78.3%)	370 (21.7%)	Azcarate-Peril et al., 2008

### RNA Extraction, Sequencing and Analysis

Cultures were grown to mid-log phase (8 h) then flash-frozen in accordance with previously used protocols (Johnson et al., 2015). Briefly, cultures were grown in MRS liquid broth at 37°C until an OD_600_ of 0.6 was reached; anaerobic samples were grown for two organisms, *L. acidophilus* and *L. amylovorus*. Media and equipment were allowed to equilibrate to anaerobic conditions in a Coy anaerobic chamber for 24 h prior to growth. RNA was extracted using the Zymo Direct-zol RNA MiniPrep kit (Zymo Research, Irvine, CA, United States) with an additional DNAse treatment and analyzed for quality using an Agilent 2100 Bioanalyzer (Agilent Technologies, Santa Clara, CA, United States). DNA library preparation and sequencing were performed by the High-throughput Sequencing and Genotyping Unit of the Roy J. Carver Biotechnology Center, University of Illinois at Urbana-Champaign, IL, United States. The Ribo-Zero bacterial kit (Illumina, San Diego, CA, United States) was used to deplete rRNAs from each sample. RNAseq libraries were prepared with the ‘Illumina TruSeq Stranded mRNAseq Sample Prep kit’ (Illumina). Libraries were then quantitated via qPCR and sequenced on one lane for 151 cycles from one end of the fragments on a HiSeq 4000 using a HiSeq 4000 sequencing kit version 1; reads were 150 nts in length. Fastq files were generated and de-multiplexed with the bcl2fastq v2.17.1.14 Conversion Software (Illumina). Geneious^®^ 11.0.2 (Kearse et al., 2012) was then used to process the reads and calculate expression levels. Reads were quality trimmed to an error probability limit of 0.001 (Phred score 20) using BBDuk (Bushnell, 2017) and filtered to remove those with less than 10 nts. Trimmed reads were mapped to their respective reference genomes using Bowtie2 (Langdon, 2015). The Geneious^®^ “Calculate Expression Level” function was used to calculate RPKM and TPM for each coding sequence; ambiguous reads were counted as partial reads. Documentation for Geneious^®^ statistical methods can be found in the software documentation^1^. Untranslated RNAs, including rRNAs, tRNAs and RNases, were not included in further analyses. Two biological replicates with two technical replicates were performed for each organism and a third previously published dataset (Johnson et al., 2015) was used as a third and independent replicate. The accession numbers of the previously published data are as follows: *L. acidophilus* (SAMN08109796), *L. amylovorus* (SAMN08109797), *L. crispatus* (SAMN08109798), *L. helveticus* (SAMN08109799), *L. gasseri* (SAMN08109801). The data generated as a part of this publication can be accessed at: *L. bulgaricus* mRNA rep1 (SAMN08564505), *L. bulgaricus* mRNA rep2 (SAMN08564506), *L. crispatus* mRNA rep1 (SAMN08564507), *L. crispatus* mRNA rep2 (SAMN08564508), *L. helveticus* mRNA rep1 (SAMN08564509), *L. helveticus* mRNA rep2 (SAMN08564510), *L. amylovorus* mRNA aerobic growth rep1 (SAMN08564511), *L. amylovorus* mRNA aerobic growth rep2 (SAMN08564512), *L. amylovorus* anaerobic growth rep1 (SAMN08564513), *L. amylovorus* mRNA anaerobic growth replicate 2 (SAMN08564514), *L. acidophilus* mRNA aerobic growth replicate 1 (SAMN08564515), *L. acidophilus* aerobic mRNA aerobic growth replicate 2 (SAMN08564516), *L. acidophilus* anaerobic growth replicate 1 (SAMN08564517), *L. acidophilus* anaerobic growth replicate 2 (SAMN08564518), *L. gasseri mRNA rep1*(SAMN08564519), *L. gasseri* mRNA rep2 (SAMN08564520).

### Statistical Analyses

All three biological replicates were used to calculate the mean RPKM and standard deviation for each coding sequence in JMP^®^ Pro, Version 11 (SAS Institute Inc., Cary, NC, United States). The RPKM and TPM values were normalized using a log_10_ transformation for correlation analyses. The 10th and 50th percentiles of the un-normalized RPKM values were used to determine the number of transcripts constituting 90 and 50% of the mRNAs in each organism. The core transcriptome was determined by detecting which core genes were present in 90% of transcripts in all six organisms. While evaluating statistical differences between the core genome and non-core genomes, means comparisons were performed using a Student’s *t*-test assuming unequal variance in JMP^®^ Pro, Version 11 (alpha 0.05). Correlation matrices analyzing several factors of the core genome, including expression levels (normalized RPKM), codon adaptation index (CAI), gene location (normalized to genome length), and GC content were generated using the corrplot (Wei and Simko, 2017) R package. Histograms were generated using JMP^®^ Pro, Version 11 (SAS Institute Inc., Cary, NC, United States).

## Results

### Genomic Arrangement of the *L. acidophilus* Complex

To better understand conserved genomic arrangement of the *L. acidophilus* complex, we used information about coding sequences that encoded mRNAs transcribed into functional proteins; non-translated RNAs, such as tRNAs, rRNAs, and RNases were excluded from our analyses. When examining only the functional coding sequences in the genomes, the number of genes per genome ranged from 1,549 in *L. bulgaricus* to 1,939 in *L. crispatus* ST1. *L. bulgaricus* ATCC 11842 possesses a large number of pseudogenes and untranslated-RNA species, which is consistent with previous reports indicating this species is undergoing genome decay (**Table 1** and **Supplementary Table S1**) (Makarova et al., 2006; Sun et al., 2015).

**FIGURE 1:**
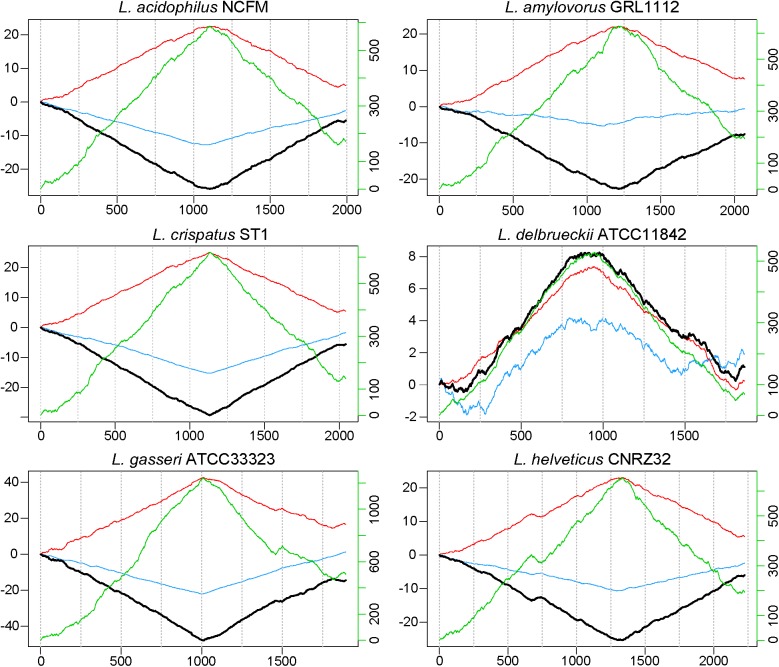
GC, TA and coding sequence skew in *L. acidophilus* complex. The cumulative skew for TA frequency (red line), GC frequency (blue line), and coding sequence density (green line) are used to predict the location of the terminus and origin (black line). The absolute minimum or maximum is predicted to be the terminus. The location in each genome (in kilobases) is displayed along the horizontal axis. The left *y*-axis shows cumulated combined skew in kb; the right *y*-axis shows cumulated coding sequence density skew.

**FIGURE 2:**
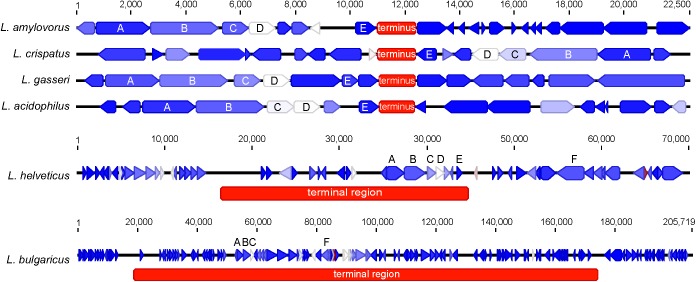
Genetic architecture of the terminus or terminal region. The genetic locus of the terminus or terminal region for each organism is displayed. The genes are colored according to their expression level on a blue-white-red gradient with blue being lowly expressed and red being highly expressed; genes are displayed as arrows. The terminus is displayed as a red box; the color of the terminus does not correspond to expression. Conserved genes across all termini are labeled as follows: **(A)** (DNA topoisomerase IV subunit B), **(B)** (DNA topoisomerase IV subunit A), **(C)** (transcriptional regulator LysR), **(D)** (manganese-dependent pyrophosphatase), **(E)** (hypothetical membrane protein), and **(F)** (carbamoyl phosphate synthetase). The scale for *L. amylovorus*, *L. crispatus, L. gasseri*, and *L. acidophilus* is displayed at the top. Individual scales for *L. helveticus* and *L. bulgaricus* are displayed above their respective figures.

When evaluating the distribution of the coding sequences on different DNA strands, we noted the entire *L. acidophilus* complex has an extremely biased coding density for the forward strand; 74–78% of coding sequences were located on the forward strand, while only 22–26% of genes were encoded on the reverse strand. Using the GC/TA skew and coding sequence strand bias, the putative terminus was predicted for each organism. The terminus is the location where the replication machinery stalls in a bacterial genome, and distinguishes the right from the left replichore. In both replichores, the leading strand is oriented from origin to terminus and the lagging strands is oriented from terminus to origin. In the *L. acidophilus* complex, all the genomes displayed a stronger TA skew than GC skew, except for *L. bulgaricus* (**Figure 1**). Some of the genomes analyzed displayed a very clear, single 2 kb region were the terminus was located. This region contained no coding sequences and the coding direction of the genes flanking the terminus distinctly switched from the right replichore to the left replichore side of the terminus. A terminus with sharp, well-defined boundaries was observed in *L. acidophilus* NCFM*, L. crispatus* ST1, *L. gasseri* ATCC 33323, and *L. amylovorus* GRL1112. In the genomes of *L. bulgaricus* ATCC 11842 and *L. helveticus* CNRZ32, there was a “terminal region,” as opposed to the single terminus seen in the other members of the complex; the “terminal region” spanned 160 kb in *L. bulgaricus* and 25 kb in *L. helveticus* (**Figures 1**, **2**). Looking at 100 genes on either side of the terminus in *L. acidophilus*, the coding bias switched from 78% of genes on the forward strand prior to the terminus to 44% of genes on the forward strand after the terminus; this strong coding switch was observed in *L. crispatus*, *L. amylovorus*, and *L. gasseri*. The coding strand bias can be seen as the green line in **Figure 1**. Unlike the clearly defined termini aforementioned, this terminal region had coding sequences almost equally coding on both strands and did not have a clear region lacking genes; regions flanking the termini in these two genomes did display a clear coding strand bias. A conserved operon was observed in all termini; all termini contained DNA topoisomerase IV subunit A followed by DNA topoisomerase IV subunit B and a manganese-dependent pyrophosphatase. All genomes except *L. bulgaricus* also contained transcriptional regulator LysR and a hypothetical membrane protein co-localized with the terminus. One gene, a carbamoyl phosphate synthetase, was conserved in the genomes with terminal regions but not found in the strong termini (**Figure 2**). Regardless of whether a single terminus or terminal region was detected in these genomes, the location of the terminus created unequal replichores causing the right replichore to always be longer than the left (**Figure 1**). Another key feature of the right replichore is the occurrence of a very dense coding region at the 3 o’clock location of the chromosome. Genes in this region also tended to have a higher GC content and codon adaptation index than in comparison to genes in the rest of the genome (**Supplementary Table S1**).

### Core Genome

**FIGURE 3:**
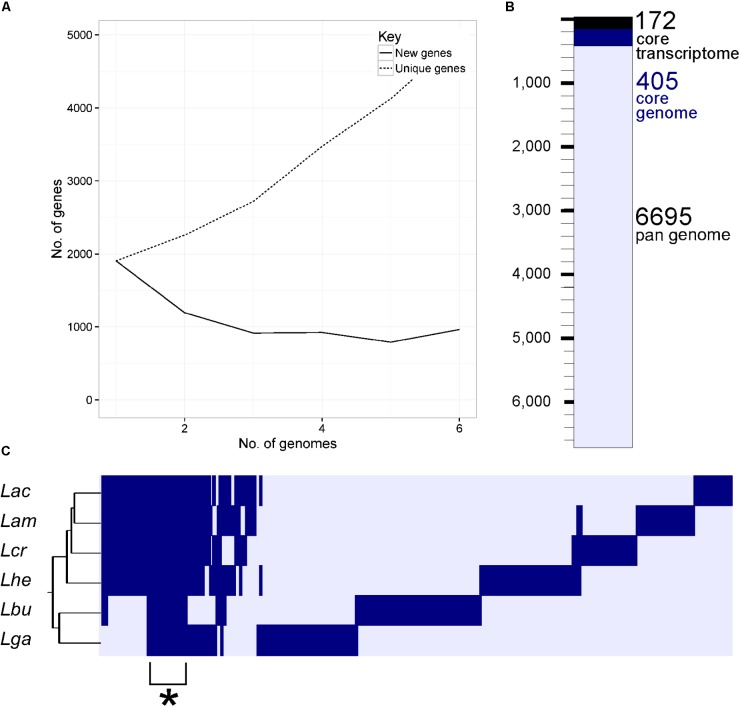
The core genome of the *L. acidophilus* complex. A rarefaction curve **(A)** demonstrates the number of new genes added by the addition of each genome (solid line) and the cumulative number of genes (dashed line) in the pan genomes as additional genomes are added to the analysis. The waterfall plot in **(B)** depicts the number of unique genes in the pan genome (light blue bar), the number of genes in the core genome (dark blue bar), and the number of genes in the core transcriptome (black bar). The core transcriptome consists of genes from the core genome that occur in the 90% of mRNA range in each organism. The cluster diagram **(C)** shows genes present (dark bar) and genes absent (light bar) in each genome; the cluster demarked with a (^∗^) is the core genome. The three letter code for each organism is as follows: Lac (*L. acidophilus*), Lam (*L. amylovorus*), Lcr (*L. crispatus*), Lhe (*L. helveticus*), Lbu (*L. bulgaricus*), and Lga (*L. gasseri*).

**FIGURE 4:**
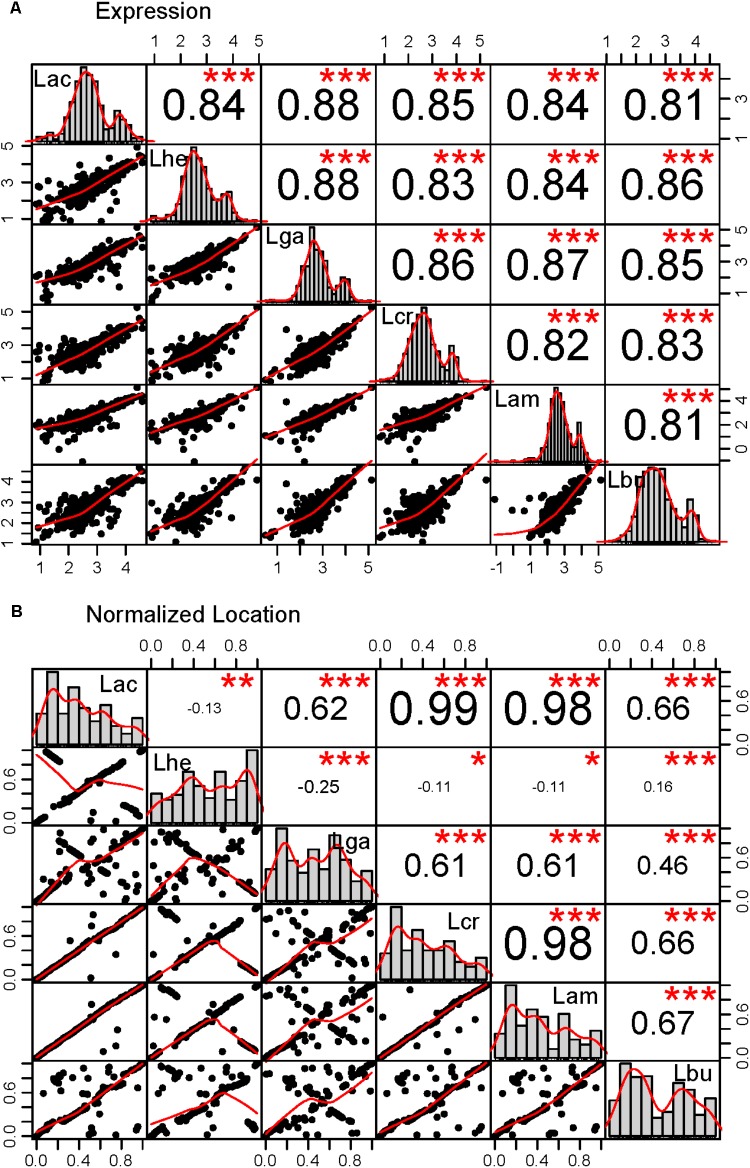
Correlations in the core genome. Correlations were performed for the core genome in all pairs of organisms in two categories: **(A)** expression as measured by the log_10_ transformed RPKM values, **(B)** location in the genome normalized to account for the length differences in the genomes. The lower left plots show the scatter plot and best fit line for each pairwise comparison. The histograms across the diagonal of each matrix show the distribution of the core genome. The *R*^2^ value for each correlation is given in the upper right boxes and the number of stars depict the statistical significance of the correlation, (^∗∗∗^*p* < 0.0001; ^∗∗^*p* < 0.001; ^∗^*p* < 0.05). The three letter code for each organism is as follows: Lac (*L. acidophilus*), Lam (*L. amylovorus*), Lcr (*L. crispatus*), Lhe (*L. helveticus*), Lbu (*L. bulgaricus*), and Lga (*L. gasseri*).

**FIGURE 5:**
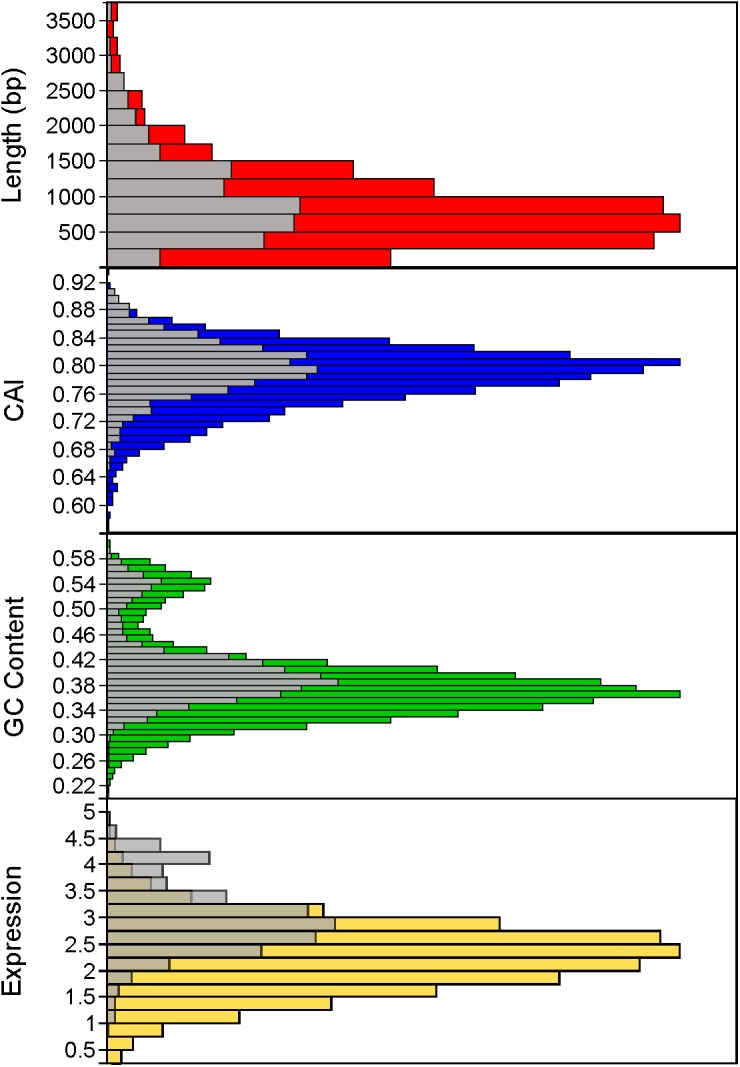
Distribution of core genome compared to non-core genome. The distribution of the core genome (gray bars) to non-core genome (colored bars) is shown for several characteristics, including gene length (in base pairs), codon adaptation index, GC content, and expression (measured by log_10_ RPKM). Histograms combine all organisms.

Using a 70% BLAST identity cutoff, we identified 405 genes that occurred in all six genomes and constituted the core genome for downstream analyses (**Figure 3** and **Supplementary Tables S2**, **S3**); the core genome is labeled using the *L. acidophilus* coding sequence gene names (i.e., LBA####). A 70% BLAST identity cutoff was used as it is known that *Lactobacillus* contains a great amount of genetic diversity, and the more permissive cutoff was used to account for this diversity. The rarefaction curve of the genomes analyzed here shows that the number of new genes is still increasing linearly and likely has not reached saturation in total number of genes in the *L. acidophilus* complex. Though there were a total of 10,494 coding sequences detected in these six genomes, there were only 6,695 unique genes in the pan genome of this complex (**Figure 3B**). *L. acidophilus* and *L. amylovorus* were the most closely related species and shared the highest number of genes, conversely, *L. bulgaricus* contained the greatest number of unique genes. We compared various genetic attributes of the core genome to the non-core genomes and saw that in all six organisms, genes in the core genome are longer and have a higher GC content (**Figures 4**, **5**, **Table 2**, and **Supplementary Figures S1**, **S2**). Codon adaption indices of the core genome demonstrate that all organisms, except *L. gasseri*, have a statistically different CAI than the non-core genome. We also analyzed expression levels of the core genome compared to the non-core genome. In all six organisms, the core genome was more highly expressed, with an average expression level around 550 RPKM, compared to 80 RPKM in the non-core. This noteworthy difference of nearly an order of magnitude suggests that core genes are significantly more highly transcribed, and more functionally critical, which is consistent with their conservation across organisms.

**Table 2:** Statistical shift in core genome.

Factor	Organism	Non-core mean	Core mean	*p*-value
Expression	*L. acidophilus*	1.97	2.782	<0.0001
	*L. amylovorus*	1.961	2.812	<0.0001
	*L. bulgaricus*	2.022	2.803	<0.0001
	*L. crispatus*	1.732	2.774	<0.0001
	*L. gasseri*	1.929	2.846	<0.0001
	*L. helveticus*	2.117	2.758	<0.0001
GC content	*L. acidophilus*	34.21%	36.94%	<0.0001
	*L. amylovorus*	38.46%	40.08%	<0.0001
	*L. bulgaricus*	50.65%	51.77%	<0.0001
	*L. crispatus*	36.84%	39.10%	<0.0001
	*L. gasseri*	34.81%	36.30%	<0.0001
	*L. helveticus*	36.21%	38.40%	<0.0001
CAI	*L. acidophilus*	0.795	0.78	<0.0001
	*L. amylovorus*	0.739	0.788	<0.0001
	*L. bulgaricus*	0.793	0.831	<0.0001
	*L. crispatus*	0.768	0.782	<0.0001
	*L. gasseri*	0.808	0.808	0.5534
	*L. helveticus*	0.799	0.805	0.0005
Length	*L. acidophilus*	930	1017	0.0102
	*L. amylovorus*	862	1019	<0.0001
	*L. bulgaricus*	867	1007	<0.0001
	*L. crispatus*	878	1019	<0.0001
	*L. gasseri*	937	1018	0.0166
	*L. helveticus*	820	1003	<0.0001

The core genome was further investigated to determine whether there are correlations with intrinsic attributes (**Figure 4** and **Table 2**). The magnitude and significance of the correlation between different genetic factors depended greatly on which organisms were being compared (**Figure 4**). *L. bulgaricus* displayed the weakest correlations with other organism across GC content and CAI, while *L. helveticus* had the lowest correlation of core genome location (**Table 2**). While overall location of the core genome did not have a strong correlation in all organisms, there were distinct clusters of genes that always co-occur. These clusters occasionally contain genes that all participate in a single common function; for example, six nucleotide metabolism proteins are contained in a single operon in all genomes (LBA1379, LBA1380, LBA1381, LBA1382, LBA1383, LBA1384). Despite the variability in the strength and magnitude of correlations in CAI, GC content and genome localization, expression was consistently highly correlated in all organisms (**Figure 4**).

### Core Transcriptome

**FIGURE 6:**
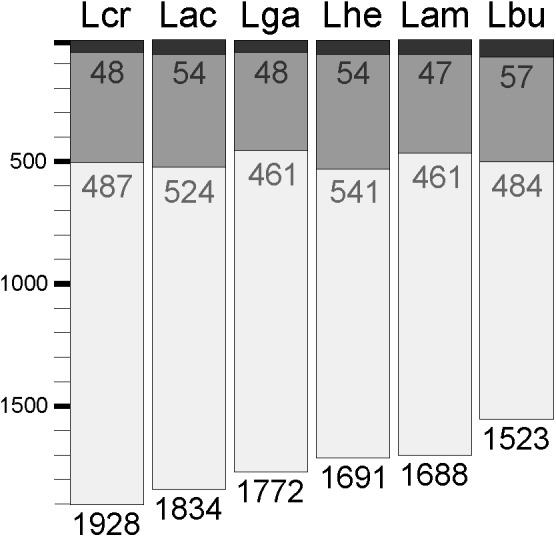
Core transcriptome of *L. acidophilus* complex. The waterfall plot of each individual organism demonstrates the total number of coding sequences in each genome (light gray bar), the number of genes that constitute 90% of the mRNAs in the cell (dark gray bar), and the number of genes that constitute 50% of the mRNAs in the cell (black bar).

**FIGURE 7:**
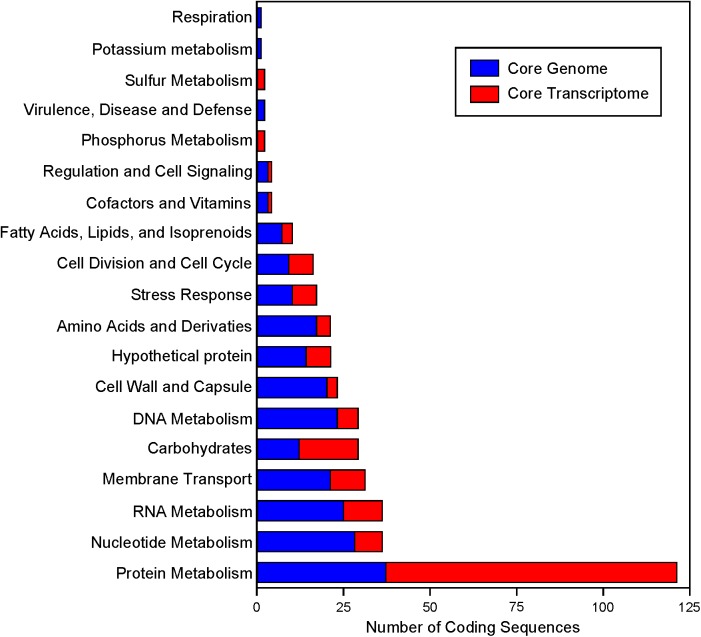
Functional annotation of the core genome and core transcriptome. RAST annotations were obtained for the core genome. The highest level functional “Category” is plotted. Genes that are part of the core genome and core transcriptome are colored in red.

**FIGURE 8:**
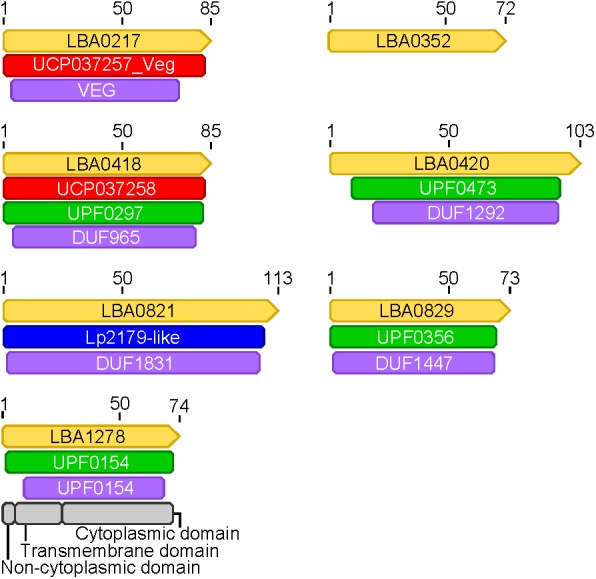
Protein domains in hypothetical core transcriptome genes. Protein domains were annotated in genes (yellow) found to be part of the core transcriptome that had hypothetical annotations. The naming convention from *L. acidophilus* is used for each gene (LBA####). Domains from different databases are colored as follows: red, green, purple, blue, gray.

Expression data from each of the samples revealed that the majority of mRNA transcripts are generated from a relatively small proportion of coding sequences. In all six organisms, 50% of the mRNAs came from 47 (*L. amylovorus*) to 57 (*L. bulgaricus*) coding sequences, less than 4% of the total genes in the entire genome (**Figure 6**). Similarly, 90% of the mRNAs were derived from approximately 500 genes, or 25–32% of the total coding sequences from each genome. Of the core genes identified, only 172 (of 405 total core genes) appear in the set of genes constituting the 90% of mRNA across all six organisms; these genes have been deemed the “core transcriptome” (**Figure 6**). There are 233 genes in the core genome that are not part of the core transcriptome. The vast majority of the core transcriptome is involved in protein metabolism (**Figure 7**), followed by carbohydrate metabolism, membrane transport, RNA metabolism, and cell division functions (**Figure 7**). There are seven proteins in the core transcriptome with a “hypothetical” annotation and unknown function based on the RAST annotation server; all of these genes code for short peptides (**Figure 8**). We searched for known protein domains in these sequences using InterproScan and determined that most of them contain conserved identifiable domains, though many are conserved domains of unknown function. A signal peptide was found on the core gene LBA1278, along with a cytoplasmic domain and transmembrane region; this gene may code for a membrane protein. The core transcriptome gene “LBA0821” contains an Lp2179-like domain that shows homology to a predicted *Lactobacillus plantarum* TATA-box binding protein, and may function as a gene regulator (**Figure 8**). The function of “hypothetical” core transcriptome genes remains largely unidentifiably from a bioinformatic standpoint.

## Discussion

Utilizing the core genome and transcriptome, we were able to shed light on some unique conserved intrinsic genetic factors in the *L. acidophilus* complex as well as identify potential novel sources of widespread regulators. While there was a great deal of conserved genetic attributes, there were notable divergent trends in a few species investigated here. While lactic acid bacteria are known for being low GC content organisms (Makarova et al., 2006; Makarova and Koonin, 2007), one species studied here, *L. delbrueckii subsp. bulgaricus,* has a very average GC content, with the ATCC 11842 strain comprising a genome wide GC content of 49.7% (**Table 1** and **Supplementary Table S1**). The five remaining species have more canonical LAB GC contents, ranging from 34.7% in *L. acidophilus* NCFM to 38.2% in *L. amylovorus* GRL 1112. The size of each genome in this study hovers around 2 Mbp, ranging from 1,864,998 bp (*L. bulgaricus* ATCC 11842) to 2,225,962 bp (*L. helveticus* CNRZ 32). There are several other LAB species beyond the *L. bulgaricus* genome studied here that diverge from the traditional GC content of lactic acid bacteria; this suggests that the low GC-content may be a key feature for discerning between particular clades within the genera constituting lactic acid bacteria, though it is not ubiquitous amongst all organisms (Kant et al., 2011; Sun et al., 2015). The preponderance of coding sequences on the forward strand suggests replication and transcription are coupled tightly within the *L. acidophilus* complex. Studies in other model organisms have suggested the purpose of strand bias may be to avoid collisions between transcription and DNA replication machinery which can stall these functions, delaying or halting cell growth (Rocha, 2008; Touchon and Rocha, 2016).

The terminus tends to be determined by GC skew in most model organisms (Frank and Lobry, 2000), however the TA skew was much stronger in this complex (**Figure 1**) and likely a result of the low GC content of these organisms. The strand coding bias and offset termini in these genomes may have implications for genetic engineering; one replichore may be replicated faster than the other, so location of gene insertions into the genomes could greatly impact transcription and efficacy of knock-ins. In the genomes that displayed terminal regions as opposed to single termini, it is not clear which strand is the leading strand and which is the lagging. It is also unclear where in the terminal region the replichores transition (**Figure 1**). The clear terminus may be an indicator of strong replication termination, while the terminal region may allow the cell to slow down or delay the replication rate by controlling the DNA synthesis rate. The lack of strand coding bias in this region may indicate that the replication machinery stalls at the flanks of the terminal region, negating the need for the strand coding biases observed throughout the rest of the genomes (**Figure 2**). Since the terminus location can only be detected in closed genomes, this study was limited to only a few published genomes. Contig ordering software uses local co-linear blocks or regions of shared sequence homology to a reference genome to assemble contigs in the correct order (Kurtz et al., 2004; Rissman et al., 2009; Galardini et al., 2015). Using information about coding strand bias and TA/GC skew may assist assemblers and contig ordering software produce higher quality draft genomes with contigs in the correct order when no reference genome is available.

The core genome is highly variable depending on the sample set used to generate the data (**Figure 3**). A previous core genome analysis in all LAB found the true core genome to be only 73 genes from 213 lactobacilli (Sun et al., 2015). All 73 of those genes were detected in our core genome. Using additional strains of each species will provide a better picture of the true pan- and core- genomes of the *L. acidophilus* complex. Previous studies have reported that the genus *Lactobacillus* contains as much genetic diversity as some phylogenetic Families (Sun et al., 2015). Using a smaller clade in the *Lactobacillus* phylogenetic tree provides a larger set of conserved genes to learn about the genetics and metabolism of a more closely related group of organisms, which gives more power behind statistical analyses and provides a broader set of sequences to compare.

As the species used here are industrially relevant given their food and human health implications, many strains from these species have been sequenced and characterized. We expect a rarefaction curve could be generated that reaches saturation, suggesting the total pan genome for this complex has been sequenced (**Figure 3**). We were surprised to see the core genome of these organisms has a higher GC content than the non-core genome (**Figure 5** and **Table 2**). The core genome is suggested to be more conserved and adapted than the pan genome (Vernikos et al., 2015), which implies that the *L. acidophilus* complex may be evolving toward a diverging GC content. We were not surprised that *L. bulgaricus* has the lowest correlation in GC content and CAI compared to the rest of the complex. *L. bulgaricus* has the highest GC content of all of the organisms investigated and is likewise the most divergent in both GC content and CAI. The species *L. delbrueckii* is known to be undergoing genome decay, a process typical of organisms that reside in nutritionally-rich environments and consequently undergo gene loss to reduce genome size by removal of expendable genes (Makarova et al., 2006; Hao et al., 2011). *L. helveticus* is known to contain a high number of transposons, causing it to have a very fluid genome (Schmid et al., 2018). This fact is validated by the low correlation of location in the core genome (**Figure 4**). Despite the lack of synteny within the core genome, the co-localization of conserved clusters suggests these may be regions that were selected for during evolution by increasing genome stability or allowing co-regulation of critical genes (**Figure 4**) (Vernikos et al., 2015). The consistency of expression level in the core genome suggests the transcription of these genes in not intrinsically determined by the genetic characteristics or adaptation of the gene itself, but rather extrinsic factors such as promoters, operon structure, or DNA structure and stereo-availability that affect the consistent transcription of the core genome.

Less than 32% of core genome coding sequences constitute the majority of mRNAs in a cell. Even fewer genes (<4% of the genome) account for the large majority (90%) of the mRNAs in a cell (**Figure 6**). This experiment only investigated expression in standard, naïve conditions. While differential RNA expression in distinct environments is well-established, future studies should focus on whether stress or different environmental conditions significantly alters the core transcriptome. Using the core transcriptome as opposed to the core genome to investigate the functional genetics of the *L. acidophilus* complex allows us to better understand the commonalities in cellular functions between these organisms. The core transcriptome is arguably more representative of the core cellular metabolic pathway conserved across different species than the core genome, as only about 40% of the core genome is highly transcribed and active in naïve growth conditions. Using transcription data in different environmental conditions in addition to genomic content will allow researchers to better understand the conservation of stress response within the *L. acidophilus* clade.

Through the use of protein domain databases, we were able to identify conserved domains in all seven hypothetical core transcriptome genes. The hypothetical core transcriptome genes are short in length, ranging from 72 to 113 amino acids, and likely under-predicted by most gene prediction software due to their small size (**Figures 7**, **8** and **Supplementary Table S2**) (Harrow et al., 2009; Edwards and Holt, 2013). Using a core genome and core transcriptome approach, we can identify small ORFs that are true protein-coding sequences and better inform gene prediction algorithms. Identifying small ORFs that are conserved across phylogenetic groups and are actively transcribed may also uncover potential novel pathways, regulation, or functions in cells. Additionally, it should be determined whether these small ORFs are truly small peptides or are the source of novel functional non-coding RNAs. Using information about conserved genetic context, shared promoters and conserved motifs across genomes may also help shed light on the activity and functionality of these ORFs. The abundance of hypothetical annotations from several annotation servers also highlights the need for database curation and propagation (Linial, 2003; McNair et al., 2018). It is known that databases used to assign gene functional annotation are often not updated nor curated with total known gene functions in the most recent literature, and can lead to an over-propagation of hypothetical and incorrect annotations (Devos and Valencia, 2001; Jones et al., 2007; Schnoes et al., 2009). Using only a handful of model organisms to assign functionality of genes based on sequence homology broadly across all domains of life is also a bioinformatics issue (Devos and Valencia, 2001). The best practice to assign true function to genes is to use taxonomic-specific databases of closely related organisms (Devos and Valencia, 2001; Brown and Sjolander, 2006). In non-model organisms, like lactobacilli, these databases are often poorly funded or non-existent. With the decrease in cost of deep sequencing and the increasing availability of data, organism-specific genetic information will hopefully be able to provide accurate functional data on many non-model organisms. This is of importance given the rising interest in diverse microbiomes and the increasing number of non-model organisms being investigated.

Understanding the conservation of genetic organization and content in a taxonomic clade is important when trying to harness organisms with biotechnological impact and potential for influencing human health. When we understand underpinning intrinsic factors in genes that affect gene maintenance and expression, we are able to better engineer organisms and select natural variants that hold the most promise of benefits to industry patients and consumers. The core transcriptome highlights the need to investigate genetic content beyond presence-absence to unveil and characterize conserved functions within cells. Investigating conserved sequences in the core genome that also appear in the core transcriptome of a taxonomic clade opens new avenues for identifying broadly applicable protein functions in cells.

## Availability of Data

The datasets analyzed in this study can be found in the Sequence Reads Archive at NCBI with the following accession numbers: *L. bulgaricus* mRNA rep1 (SAMN08564505), *L. bulgaricus* mRNA rep2 (SAMN08564506), *L. crispatus* mRNA rep1 (SAMN08564507), *L. crispatus* mRNA rep2 (SAMN08564508), *L. helveticus* mRNA rep1 (SAMN08564509), *L. helveticus* mRNA rep2 (SAMN08564510), *L. amylovorus* mRNA aerobic growth rep1 (SAMN08564511), *L. amylovorus* mRNA aerobic growth rep2 (SAMN08564512), *L. amylovorus* anaerobic growth rep1 (SAMN08564513), *L. amylovorus* mRNA anaerobic growth replicate 2 (SAMN08564514), *L. acidophilus* mRNA aerobic growth replicate 1 (SAMN08564515), *L. acidophilus* aerobic mRNA aerobic growth replicate 2 (SAMN08564516), *L. acidophilus* anaerobic growth replicate 1 (SAMN08564517), *L. acidophilus* anaerobic growth replicate 2 (SAMN08564518), *L. gasseri mRNA rep1*(SAMN08564519), and *L. gasseri* mRNA rep2 (SAMN08564520).

## Author Contributions

AC and RB planned the work and interpreted results. AC performed the analyses and wrote the manuscript.

## Conflict of Interest Statement

The authors declare that the research was conducted in the absence of any commercial or financial relationships that could be construed as a potential conflict of interest.

## Footnotes

1 https://assets.geneious.com/manual/11.0/GeneiousManualsu97.html

## Supplementary Material

FIGURE S1 Core genome correlation in GC content and CAI. Correlations were performed for all pairs of organisms in two categories: GC content and codon adaptation index (CAI) calculated with each organism’s core genome as the training set. The lower left plots show the scatter plot and best fit line for each pairwise comparison. The histograms across the diagonal of each matrix show the distribution of the core genome. The *R*^2^ value for each correlation is given in the upper right boxes and the number of stars depict the statistical significance of the correlation, (^∗∗∗^*p* < 0.0001; ^∗∗^*p* < 0.001; ^∗^*p* < 0.05).

FIGURE S2 Distribution of core genome compared to non-core genome by organism. The distribution of the core genome (gray bars) to non-core genome (colored bars) is shown for several characteristics, including gene length (in base pairs), codon adaptation index, GC content, and expression (measured by log_10_ RPKM).

TABLE S1 All coding sequences from *L. acidophilus* complex.

TABLE S2 Core genome functional annotation.

TABLE S3 Gene presence-absence in pan genome.

## References

[B1] Altermann E. Russell W. M. Azcarate-Peril M. A. Barrangou R. Buck B. L. McAuliffe O. (2005). Complete genome sequence of the probiotic lactic acid bacterium *Lactobacillus acidophilus* NCFM. *Proc. Natl. Acad. Sci. U.S.A.* 102 3906–3912. 10.1073/pnas.0409188102 15671160 PMC554803

[B2] Axelsson L. (2004). *Lactic Acid Bacteria: Classification and Physiology.* New York, NY: Marcel Dekker Inc. 10.1201/9780824752033.ch1

[B3] Azcarate-Peril M. A. Altermann E. Goh Y. J. Tallon R. Sanozky-Dawes R. B. Pfeiler E. A. (2008). Analysis of the genome sequence of *Lactobacillus gasseri* ATCC 33323 reveals the molecular basis of an autochthonous intestinal organism. *Appl. Environ. Microbiol.* 74 4610–4625. 10.1128/AEM.00054-08 18539810 PMC2519322

[B4] Aziz R. K. Bartels D. Best A. A. Dejongh M. Disz T. Edwards R. A. (2008). The RAST Server: rapid annotations using subsystems technology. *BMC Genomics* 9:75. 10.1186/1471-2164-9-75 18261238 PMC2265698

[B5] Bernardeau M. Guguen M. Vernoux J. P. (2006). Beneficial lactobacilli in food and feed: long-term use, biodiversity and proposals for specific and realistic safety assessments. *FEMS Microbiol. Rev.* 30 487–513. 10.1111/j.1574-6976.2006.00020.x 16774584

[B6] Bouyioukos C. Elati M. Kepes F. (2016). Analysis tools for the interplay between genome layout and regulation. *BMC Bioinformatics* 17(Suppl. 5):191. 10.1186/s12859-016-1047-0 27294345 PMC4905612

[B7] Broadbent J. R. Hughes J. E. Welker D. L. Tompkins T. A. Steele J. L. (2013). Complete genome sequence for *Lactobacillus helveticus* CNRZ 32 an industrial cheese starter and cheese flavor adjunct. *Genome Announc.* 1:e00590-13. 10.1128/genomeA.00590-13 23969047 PMC3751602

[B8] Brown D. Sjolander K. (2006). Functional classification using phylogenomic inference. *PLoS Comput. Biol.* 2:e77. 10.1371/journal.pcbi.0020077 16846248 PMC1484587

[B9] Bushnell B. (2017). *BBDuk* *Trimmer* Available at: http://jgi.doe.gov/data-and-tools/bb-tools/

[B10] Canchaya C. Claesson M. J. Fitzgerald G. F. Van Sinderen D. O’toole P. W. (2006). Diversity of the genus *Lactobacillus* revealed by comparative genomics of five species. *Microbiology* 152 3185–3196. 10.1099/mic.0.29140-0 17074890

[B11] Cato E. P. Moore W. E. C. Johnson J. L. (1983). Synonymy of strains of *“Lactobacillus acidophilus”* group A2 (Johnson et al. 1980) with the type strain of *Lactobacillus crispatus* (Brygoo and Aladame 1953) Moore and Holdeman 1970. *Int. J. Syst. Bacteriol.* 33 426–428. 10.1099/00207713-33-2-426

[B12] Devos D. Valencia A. (2001). Intrinsic errors in genome annotation. *Trends Genet.* 17 429–431. 10.1016/S0168-9525(01)02348-4 11485799

[B13] Edwards D. J. Holt K. E. (2013). Beginner’s guide to comparative bacterial genome analysis using next-generation sequence data. *Microb. Inform. Exp.* 3:2. 10.1186/2042-5783-3-2 23575213 PMC3630013

[B14] Frank A. C. Lobry J. R. (2000). Oriloc: prediction of replication boundaries in unannotated bacterial chromosomes. *Bioinformatics* 16 560–561. 10.1093/bioinformatics/16.6.560 10980154

[B15] Galardini M. Mengoni A. Bazzicalupo M. (2015). Mapping contigs using CONTIGuator. *Methods Mol. Biol.* 1231 163–176. 10.1007/978-1-4939-1720-4_11 25343865

[B16] Hao P. Zheng H. Yu Y. Ding G. Gu W. Chen S. (2011). Complete sequencing and pan-genomic analysis of *Lactobacillus delbrueckii* subsp. *bulgaricus* reveal its genetic basis for industrial yogurt production. *PLoS One* 6:e15964. 10.1371/journal.pone.0015964 21264216 PMC3022021

[B17] Harrow J. Nagy A. Reymond A. Alioto T. Patthy L. Antonarakis S. E. (2009). Identifying protein-coding genes in genomic sequences. *Genome Biol.* 10:201. 10.1186/gb-2009-10-1-201 19226436 PMC2687780

[B18] Huynen M. Snel B. Lathe W. III Bork P. (2000). Predicting protein function by genomic context: quantitative evaluation and qualitative inferences. *Genome Res.* 10 1204–1210. 10.1101/gr.10.8.1204 10958638 PMC310926

[B19] Huynen M. A. Snel B. (2000). Gene and context: integrative approaches to genome analysis. *Adv. Protein Chem.* 54 345–379. 10.1016/S0065-3233(00)54010-8 10829232

[B20] Johnson B. R. Hymes J. Sanozky-Dawes R. Henriksen E. D. Barrangou R. Klaenhammer T. R. (2015). Conserved S-layer-associated proteins revealed by exoproteomic survey of S-layer-forming lactobacilli. *Appl. Environ. Microbiol.* 82 134–145. 10.1128/AEM.01968-15 26475115 PMC4702614

[B21] Jones C. E. Brown A. L. Baumann U. (2007). Estimating the annotation error rate of curated GO database sequence annotations. *BMC Bioinformatics* 8:170. 10.1186/1471-2105-8-170 17519041 PMC1892569

[B22] Kandler O. Weiss N. (1986). *Genus Lactobacillus.* Baltimore, MD: Williams and Wilkins.

[B23] Kant R. Blom J. Palva A. Siezen R. J. De Vos W. M. (2011). Comparative genomics of Lactobacillus. *Microb. Biotechnol.* 4 323–332. 10.1111/j.1751-7915.2010.00215.x 21375712 PMC3818991

[B24] Kearse M. Moir R. Wilson A. Stones-Havas S. Cheung M. Sturrock S. (2012). Geneious Basic: an integrated and extendable desktop software platform for the organization and analysis of sequence data. *Bioinformatics* 28 1647–1649. 10.1093/bioinformatics/bts199 22543367 PMC3371832

[B25] Kilian M. (2005). *Streptococcus and Lactobacillus.* London: Hodder Arnold.

[B26] Klaenhammer T. R. Kleerebezem M. Kopp M. V. Rescigno M. (2012). The impact of probiotics and prebiotics on the immune system. *Nat. Rev. Immunol.* 12 728–734. 10.1038/nri3312 23007572

[B27] Kullen M. J. Sanozky-Dawes R. B. Crowell D. C. Klaenhammer T. R. (2001). Use of the DNA sequence of variable regions of the 16S rRNA gene for rapid and accurate identification of bacteria in the *Lactobacillus acidophilus* complex. *J. Appl. Microbiol.* 89 511–516. 10.1046/j.1365-2672.2000.01146.x 11021584

[B28] Kurtz S. Phillippy A. Delcher A. L. Smoot M. Shumway M. Antonescu C. (2004). Versatile and open software for comparing large genomes. *Genome Biol.* 5:R12. 10.1186/gb-2004-5-2-r12 14759262 PMC395750

[B29] Langdon W. B. (2015). Performance of genetic programming optimised Bowtie2 on genome comparison and analytic testing (GCAT) benchmarks. *Biodata Min.* 8:1. 10.1186/s13040-014-0034-0 25621011 PMC4304608

[B30] Linial M. (2003). How incorrect annotations evolve–the case of short ORFs. *Trends Biotechnol.* 21 298–300. 10.1016/S0167-7799(03)00139-2 12837613

[B31] Lobry J. R. (1996). Asymmetric substitution patterns in the two DNA strands of bacteria. *Mol. Biol. Evol.* 13 660–665. 10.1093/oxfordjournals.molbev.a025626 8676740

[B32] Lobry J. R. Lobry C. (1999). Evolution of DNA base composition under no-strand-bias conditions when the substitution rates are not constant. *Mol. Biol. Evol.* 16 719–723. 10.1093/oxfordjournals.molbev.a026156 10368950

[B33] Makarova K. Slesarev A. Wolf Y. Sorokin A. Mirkin B. Koonin E. (2006). Comparative genomics of the lactic acid bacteria. *Proc. Natl. Acad. Sci. U.S.A.* 103 15611–15616. 10.1073/pnas.0607117103 17030793 PMC1622870

[B34] Makarova K. S. Koonin E. V. (2007). Evolutionary genomics of lactic acid bacteria. *J. Bacteriol.* 189 1199–1208. 10.1128/JB.01351-06 17085562 PMC1797341

[B35] McNair K. Aziz R. K. Pusch G. D. Overbeek R. Dutilh B. E. Edwards R. (2018). Phage genome annotation using the RAST pipeline. *Methods Mol. Biol.* 1681 231–238. 10.1007/978-1-4939-7343-9_17 29134599

[B36] Nakamura L. K. (1981). *Lactobacillus amylovorus*, a new starch-hydrolyzing species from cattle waste-corn fermentations. *Int. J. Syst. Bacteriol.* 31 56–63. 10.1099/00207713-31-1-56

[B37] Ojala T. Kuparinen V. Koskinen J. P. Alatalo E. Holm L. Auvinen P. (2010). Genome sequence of *Lactobacillus crispatus* ST1. *J. Bacteriol.* 192 3547–3548. 10.1128/JB.00399-10 20435723 PMC2897677

[B38] Page A. J. Cummins C. A. Hunt M. Wong V. K. Reuter S. Holden M. T. (2015). Roary: rapid large-scale prokaryote pan genome analysis. *Bioinformatics* 31 3691–3693. 10.1093/bioinformatics/btv421 26198102 PMC4817141

[B39] R Core Team (2017). *R: A Language and Environment for Statistical Computing.* Vienna: R Foundation for Statistical Computing.

[B40] Rice P. Longden I. Bleasby A. (2000). EMBOSS: the European molecular biology open software suite. *Trends Genet.* 16 276–277. 10.1016/S0168-9525(00)02024-2 10827456

[B41] Rissman A. I. Mau B. Biehl B. S. Darling A. E. Glasner J. D. Perna N. T. (2009). Reordering contigs of draft genomes using the Mauve aligner. *Bioinformatics* 25 2071–2073. 10.1093/bioinformatics/btp356 19515959 PMC2723005

[B42] Rocha E. P. (2008). The organization of the bacterial genome. *Annu. Rev. Genet.* 42 211–233. 10.1146/annurev.genet.42.110807.091653 18605898

[B43] Schmid M. Muri J. Melidis D. Varadarajan A. R. Somerville V. Wicki A. (2018). Comparative genomics of completely sequenced *Lactobacillus helveticus* genomes provides insights into strain-specific genes and resolves metagenomics data down to the strain level. *Front. Microbiol.* 9:63. 10.3389/fmicb.2018.00063 29441050 PMC5797582

[B44] Schnoes A. M. Brown S. D. Dodevski I. Babbitt P. C. (2009). Annotation error in public databases: misannotation of molecular function in enzyme superfamilies. *PLoS Comput. Biol.* 5:e1000605. 10.1371/journal.pcbi.1000605 20011109 PMC2781113

[B45] Seemann T. (2014). Prokka: rapid prokaryotic genome annotation. *Bioinformatics* 30 2068–2069. 10.1093/bioinformatics/btu153 24642063

[B46] Sun Z. Harris H. M. Mccann A. Guo C. Argimon S. Zhang W. (2015). Expanding the biotechnology potential of lactobacilli through comparative genomics of 213 strains and associated genera. *Nat. Commun.* 6:8322. 10.1038/ncomms9322 26415554 PMC4667430

[B47] Thompson K. Collins M. (1991). Molecular cloning in *Lactobacillus helveticus* by plasmid pSA3::pVA797 co-integrate formation and conjugal transfer. *Appl. Microbiol. Biotechnol.* 35 334–338. 10.1007/BF00172722 1367540

[B48] Touchon M. Rocha E. P. (2016). Coevolution of the organization and structure of prokaryotic genomes. *Cold Spring Harb. Perspect. Biol.* 8:a018168. 10.1101/cshperspect.a018168 26729648 PMC4691797

[B49] Vernikos G. Medini D. Riley D. R. Tettelin H. (2015). Ten years of pan-genome analyses. *Curr. Opin. Microbiol.* 23 148–154. 10.1016/j.mib.2014.11.016 25483351

[B50] Wei T. Simko V. (2017). *R Package ”Corrplot”: Visualization of a Correlation Matrix.* Available at: https://github.com/taiyun/corrplot

