# Supplementary figures and images for: Conserved Genome Organization and Core Transcriptome of the Lactobacillus acidophilus Complex
Source: Front Microbiol. 2018 Aug 13;9:1834. doi: 10.3389/fmicb.2018.01834 (PMC6099100; doi:10.3389/fmicb.2018.01834)

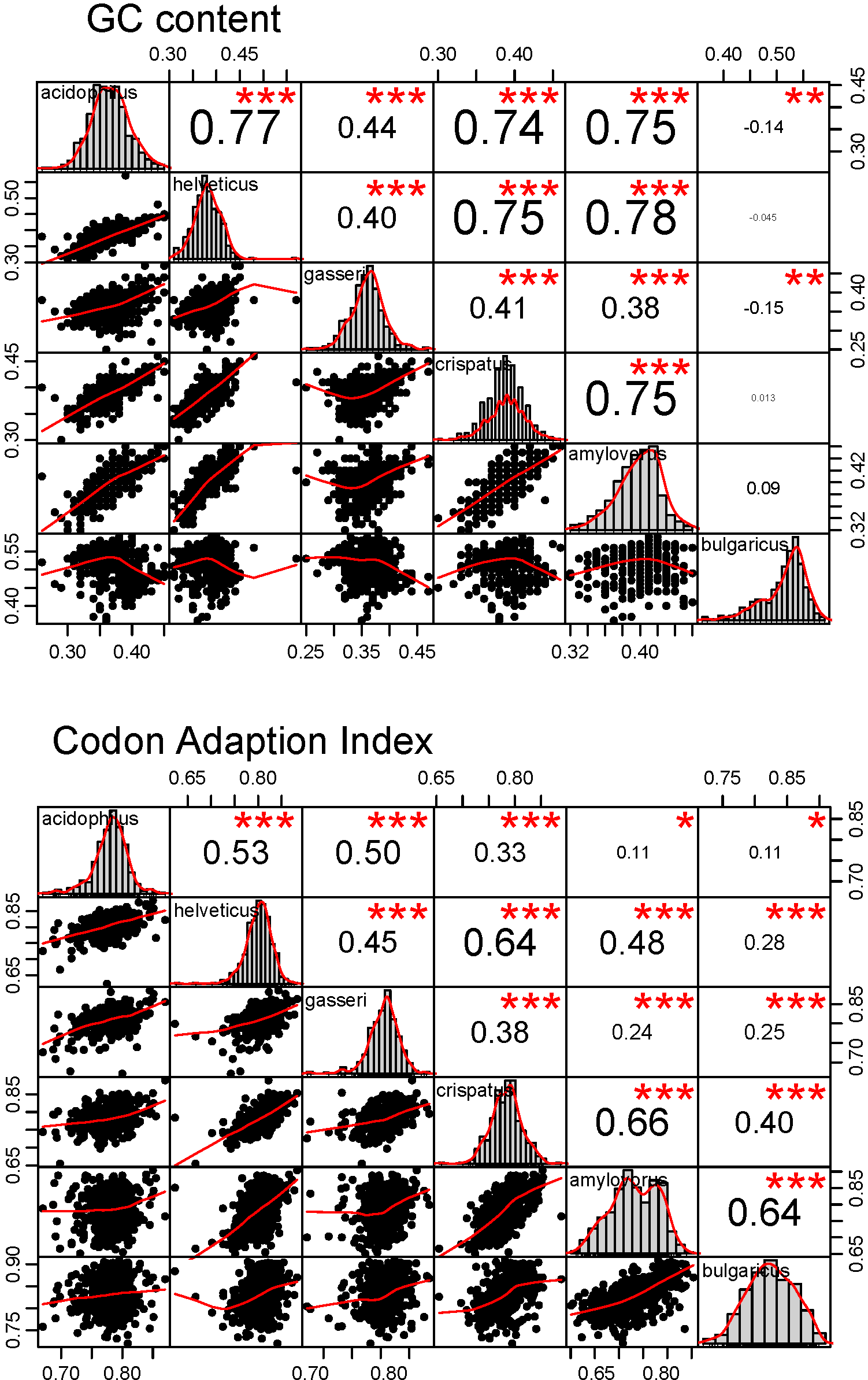

Supplement: FIGURE S1 — Core genome correlation in GC content and CAI. Correlations were performed for all pairs of organisms in two categories: GC content and codon adaptation index (CAI) calculated with each organism’s core genome as the training set. The lower left plots show the scatter plot and best fit line for each pairwise comparison. The histograms across the diagonal of each matrix show the distribution of the core genome. The R2 value for each correlation is given in the upper right boxes and the number of stars depict the statistical significance of the correlation, (∗∗∗p < 0.0001; ∗∗p < 0.001; ∗p < 0.05). [file Image_1.TIFF]

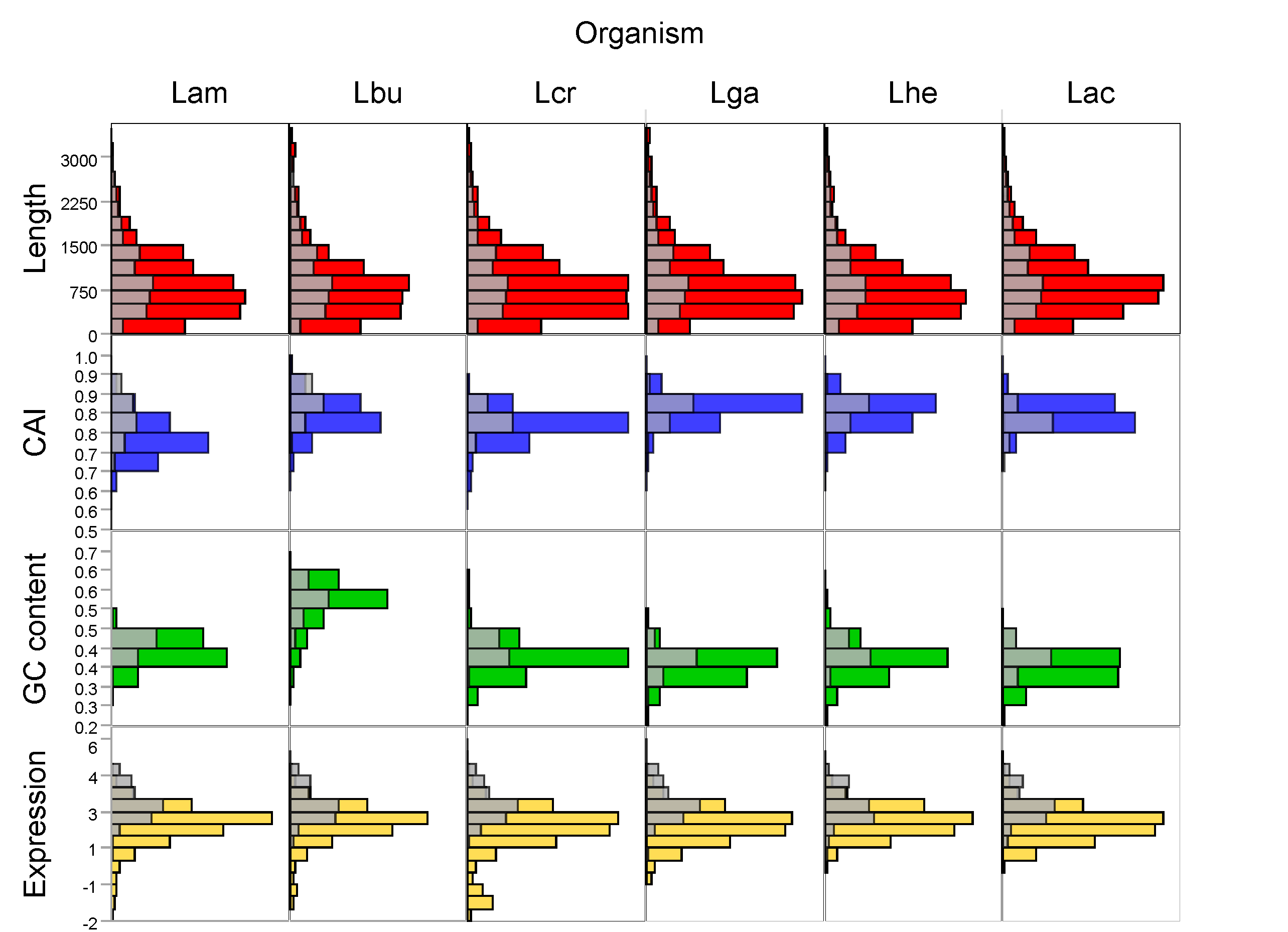

Supplement: FIGURE S2 — Distribution of core genome compared to non-core genome by organism. The distribution of the core genome (gray bars) to non-core genome (colored bars) is shown for several characteristics, including gene length (in base pairs), codon adaptation index, GC content, and expression (measured by log10 RPKM). [file Image_2.TIFF]
